# Supplementary material for: Acceptability of HIV self-testing among HIV high-risk Adolescent Girls and Young Women (AGYW) in urban settings in Uganda
Source: PLOS Glob Public Health. 2026 Feb 23;6(2):e0005857. doi: 10.1371/journal.pgph.0005857 (PMC12928388; doi:10.1371/journal.pgph.0005857)
Supplement: S1 File — (PDF) [file pgph.0005857.s001.pdf]

Questionnaire

Study tittle: Barriers and facilitators of intention to use, and the preferred model for HIV Self-Testing among young women in the Kampala Metropolitan Area

Participant ID: |_|.|_|.|_| Current

Physical Address:

District: |________________________|

Division/Municipality: |_________________________|

Village/Zone: |_________________________|

Part A: Participant demographic characteristics

1. What is your current age in completed years? |___|.|___|
2. What is your current marital status?
    1. |___| Single
    2. |___| Married
    3. |___| Cohabiting
    4. |___| Divorced
    5. |___| Widowed
    6. |___| Separated

3. What is your religion?

    1. |___| Anglican
    2. |___| Catholic
    3. |___| Moslem
    4. |___| Seventh Day Adventist
    5. |___| Pentecostal
    6. |___| None
    7. |___| Others |___________________|

4. Are you currents in school?

    1. |___| Yes
    2. |___| No

5. What is your highest level of education?

1. |___| None
2. |___| Primary level
3. |___| Secondary level
4. |___| Advance secondary level
5. |___| Tertiary level (university/tertiary institutions)

6. Do you currently have a job?
    1. |___| Yes
    2. |___| No
7. If Yes to 6 above, what job do you do?

    |___________________________|

8. On average, how much income do you generate per month?

|___||___|,|___||___||___|,|___||___||___|

Part B: Individual/behavioral characteristics

9. Have you ever been pregnant?
    1. |___| Yes
    2. |___| No

If No go to Question 12

10. What is the current parity? |________________|
11. How many live biological children do you have? |___|.|___|
12. Do you have a desire to get pregnant in the next 12 months?
    1. |___| Yes
    2. |___| No
13. Have you used any of the modern contraceptives in the past 6 months?
    1. |___| Yes
    2. |___| No

If "No" go to question15

14. If yes to question 11, which Contraceptive did you use?

1. |___| Oral pills
2. |___| Injectable
3. |___| Implant
4. |___| Intrauterine device
5. |___| Condoms
6. |___| Others |__________________|

15. Have you had sexual intercourse with a sexual partner who is not your primary partner in the past 6 months?

1. |___| Yes
2. |___| No

16. How many other sexual partners have you had in the past 6 months apart from the primary partner? |___|.|___|

17. Have you had anal sex in the past 6 months?

1. |___| Yes
2. |___| No

18. Have you had any sexual intercourse in exchange for money or any other gifts in the past 6 months?

1. |___| Yes
2. |___| No

19. Have you had an abnormal vaginal discharge or a diagnosed sexually transmitted infection in the past 6 months?

1. |___| Yes
2. |___| No

20. Have you taken HIV post exposure prophylaxis (medicine taken by an individual after being exposed to a potential source of HIV) in the past 12 months?

1. |___| Yes
2. |___| No

21. Have you used condom in the most recent sexual intercourse?

1. |___| Yes
2. |___| No

22. Have you used Alcohol just before a sexual intercourse in the past 6 months?

1. |___| Yes
2. |___| No

23. On a scale of 0 to 10, where 0 means “no risk of getting HIV” and 10 means “a very high risk of getting HIV”, how would you grade your risk of getting HIV in the past 6 months? |___|.|___|

Part C: Partner information

24. How old is your primary partner? |___|.|___|

25. How many of the other sexual partners in the past 6 months are older than you by 5 or more years? |___|.|___|

26. Have you ever faced sexual violence from any other person in the past 12 months?

1. |___| Yes
2. |___| No

27. Have you faced any violence from any of the intimate partner(s) in the past 12 months?

1. |___| Yes
2. |___| No

Part D: Information on HIV testing and HIV self-testing services

28. Have you ever tested for HIV in your lifetime?

1. |___| Yes
2. |___| No

If “No” go to question 31

29. Have you ever tested with any of your sexual partners?

1. |___| Yes
2. |___| No

30. Have you tested for HIV in the past 6 months?

1. |___| Yes

2. |___| No

31. Have you ever heard about HIV self-testing?

1. |___| Yes
2. |___| No

If No, go to question 34

32. Do you know how to use any of the available HIVST kits?

1. |___| Yes
2. |___| No

33. Have you ever used an HIVST kit to test yourself for HIV?

1. |___| Yes
2. |___| No

Part D: intention to use HIVST services

Affective attitude

34. How much do you agree or disagree that HIV self-testing is a comfortable/good idea for young women like you?

1.|___|Strongly disagree

2.|___|Disagree

3.|___|Neutral

4.|___|Agree

5.|___|Strongly agree

Burden

35. How much do you agree or disagree that using HIV self-testing kits seems like an easy process to you?

1.|___|Strongly disagree

2.|___|Disagree

3.|___|Neutral

4.|___|Agree

5.|___|Strongly agree

Self-efficacy

36. How confident are you in your ability to use HIV self-testing properly and interpret the results?

1. |___|Strongly not confident

2. |___|Not confident

3.|___|Neutral

4. |___|Confident

5. |___|Strongly confident

Ethicality

37. How much do you agree or disagree that the use of HIV self-testing aligns with your beliefs and values?

1.|___|Strongly disagree

2.|___|Disagree

3.|___|Neutral

4.|___|Agree

5.|___|Strongly agree

Opportunity Costs

38. To what extent do you agree or disagree that you would give up some of your time money or other priorities to use the HIV self-testing services?

1.|___|Strongly disagree

2.|___|Disagree

3.|___|Neutral

4.|___|Agree

5.|___|Strongly agree

Perceived effectiveness

39. To what extent do you agree or disagree that HIV self-testing would meaningfully help you to know your HIV status?

1.|___|Strongly disagree

2.|___|Disagree

3.|___|Neutral

4.|___|Agree

5.|___|Strongly agree

Coherence

40. To what extent do you agree or disagree that you know HIV self-testing can work for young women like you to be aware of your HIV status?

1.|___|Strongly disagree

2.|___|Disagree

3.|___|Neutral

4.|___|Agree

5.|___|Strongly agree

41. If the HIVST services were available to you, on a scale of 0 to 10, where 0 means you can never use them and 10 means you will most definitely use them, how likely would you plan or be willing to use HIVST services? |___|.|___|

Part E: Health system related information

42. If you were to be given HIVST kits for free, would you like to pick it yourself from a distribution center or pick it through a social contact like a peer or a community health worker?

1. |___| Pick it myself at health facility
2. |___| Pick it myself from a community distribution center
3. |___| Pick it through a social contact (like peer, CHW, VHT)
4. |___| None of the above, rather |_____________________|
5. |___| Not sure

43. If you were to carry out HIV testing on yourself using a self-test kit, which type of test kit would you prefer?

1. |___| Oral test kit
2. |___| Blood-based kit
3. |___| Not sure

44. If you were to carry out HIV testing on yourself using a self-test kit, would you need help from any other person like a peer/ VHT/health worker?

1. |___| Yes
2. |___| No
3. |___| Not sure

45. If you were to carry out HIV testing on yourself using a self-test kit, would you need to get counseling before or after testing from a health care worker?

1. |___| Yes

2. |___| No

3. |___| Not sure

46. Have you ever looked for an HIV self-test kit at your nearest health facility and failed to get it?

1. |___| Yes

2. |___| No

47. For the last time you had an HIV test at a health facility or community center did the health care worker show you a good attitude while receiving the service?

1. |___| Yes

2. |___| No

3. |___| I don't remember

48. The average cost of HIV self-testing kits in Kampala Metropolitan Area is about Ugx. 25000/=. At this cost, would you be willing to buy and used HIV self-test kits to test yourself?

1. |___| Yes

2. |___| No

49. What is the minimum amount of money in Uganda shillings would you be willing to spend on an HIV self-test kits for testing yourself at least once?

|___|,|___||___||___|,|___||___||___|

50. What do you think should be the ideal cost of an HIV self-testing kit?

|___|,|___||___||___|,|___||___||___|

51. What is the maximum amount of money in Uganda shillings would you be willing to spend on an HIV self-test kits for testing yourself at least once?

|___|,|___||___||___|,|___||___||___|
