# Supplementary material for: Acceptability of HIV self-testing among HIV high-risk Adolescent Girls and Young Women (AGYW) in urban settings in Uganda
Source: PLOS Glob Public Health. 2026 Feb 23;6(2):e0005857. doi: 10.1371/journal.pgph.0005857 (PMC12928388; doi:10.1371/journal.pgph.0005857)
Supplement: Attachment — Submitted filename: Response to Reviewer comments.pdf [file pgph.0005857.s003.pdf]

Manuscript title: Acceptability of HIV self-testing among HIV high-risk Adolescent Girls and Young Women (AGYW) in urban settings in Uganda

Manuscript Number: PGPH-D-25-01949

Dear Editor,

Following the review of the above mentioned manuscript, the reviewers raised some comments to address.

We have addressed these comments point by point as described below (responses are in bold-italics). The cited line numbers correspond to the Revised Manuscript with tracked changes

Reviewer #1: This manuscript presents an important topic on the acceptability of HIV self-testing services. Overall, the manuscript is well organised and written clearly. I have some suggestions to improve the manuscript:

Major revisions:
1. Please expand lines 84-85 to include that you also explored which behavioural and clinical factors are associated with acceptability of HIVST.

The objective was expanded to reflect assessment of factors associated with acceptability of HIVST (see line 88)

2. Can the authors expand more on VHTs?
2.1. What is the composition (sex and age) of these VHTs?
2.2. How many teams were used in this study?
2.3. Were VHTs provided with any training? What was provided as part of training?

The information about who VHTs are, was included see line 126-131

3. Dependent variable:
3.1. While the composite scoring and median split provide a simple way to classify participants, this method risks information loss and relies on an arbitrary cut-off. A more robust approach would be to analyse the summed TFA score as a continuous outcome, which preserves variability and statistical power. However, it seems that a binary definition of acceptability is desired for interpretability; as such, I suggest conducting sensitivity analyses with multiple thresholds (e.g., >28, >35) to show robustness.

Whereas analyzing summed up TFA scores would be the best option, the continuous outcome was not normally distributed, which violated a key

assumption of Linear regression for the case of analysis factors associated with acceptability of HIVST. Categorizing the outcome as a binary outcome (though risks information loss) using the median of possible scores leverages the important feature of median which divides the distribution by half. Moreover, in the results we provide a summary of the total scores using median, interquartile range and the range of acceptability scores (summed up scores) to guide readers on evaluating their distribution (see line 228)

4. Line 149: "...having a sexually transmitted disease in the past six months defined as having had a diagnosis or an abnormal vaginal discharge..." This is a bit awkward, as one is a confirmed condition, while the other is a non-specific symptom. Can you please clarify whether those with abnormal vaginal discharge were treated for STIs, i.e., syndromic management, and thus were classified as having an STI?

Whereas presence of abnormal vaginal discharge doesn't necessarily mean having a sexually transmitted infection (STI) (and neither does its absence mean absence of STI), it has been used in our setting as a proxy for suspecting STI. Furthermore, given the sub-optimal health seeking behaviors, we used this subjective proxy in this HIV high-risk and sexually active sub-population. We however, acknowledge that this may bring about misclassification and we have added it in the limitation section (see line 375-379).

5. Line 210-212: Acceptability of HIVST among AGYW:
5.1. Since TFA is multidimensional, authors could also report construct-specific scores (e.g., mean Likert scores for affective attitude, burden, self-efficacy, etc.). Perhaps provide a table or a figure.

We acknowledge and appreciate the guidance, however, our main aim was to look at acceptability as a whole in this study. Moreover, further construct validation through exploratory factor analysis conducted as an extension to this work confirmed that acceptability as measured using the TFA in our population yielded a unidimensional structure, implying that the constructs collectively measured a single outcome of acceptability.

6. In table 3: Please add a column to show the number and percentages of those willing to use HIVST services for each variable/category.

A column that shows the proportion/percentage of those willing to use HIVST for each independent variable was added to table 3

7. Line 250: Given the sampling technique, which is a non-probability method, i.e., the sample was not randomly selected from the target population, generalisability might not

hold. As such, I suggest rephrasing "… acceptability of HIVST in our population…" to say "in our study".

Thank you. "our population" has been changed to "our study" (see line 268)

8. Lines 356-360 Conclusion:
8.1. The conclusion seems to overstate the findings relative to the study design and sample. Specifically, the statement that HIVST is "highly acceptable among HIV high-risk AGYW in urban settings" and the suggestion to scale up services imply broader generalisability and causal evidence that the study design does not support. I recommend revising the conclusion to clarify that acceptability was observed within the enrolled sample.

The conclusion has been revised to reflect the limited generalizability. Furthermore, we acknowledge this limitation in the Limitation section (line 382-386)

8.2. Scale-up recommendations should be framed cautiously, while still noting the potential value of integrating behavioural and psychological considerations, e.g., future implementation efforts could consider strategies addressing both behavioural risks and psychological vulnerabilities to optimise uptake and impact.

Scale-up recommendation has been rephrased (see line 384-386)

8.3. It would be helpful to mention that generalisability is limited by the use of consecutive sampling and the urban-specific study context and that further research is needed to confirm acceptability in other populations and settings.

This limitation is acknowledged in the limitation section (line 382-386)

Minor revisions:
1. I am not familiar with the KoboCollect toolkit. So, perhaps if wording allows, authors can mention what this tool is or provide any reference.

The KoboCollect toolkit is an open source data management software that was used. The link to the website has been provided in the section of "data management" (see line 140)

2. Line 149: rephrase "sexually transmitted disease" to "sexually transmitted infection"

"sexually transmitted disease" has been rephrased to "sexually transmitted infection" (see line 163)

3. Line 164-165: “At unadjusted analysis the crude prevalence ratios (cPR) were computed, their 95% CIs and P values were computed.” I suggest revising to avoid repetition: At unadjusted analysis the crude prevalence ratios (cPR) and their 95% CIs and P values were computed.

Statement rephrased to eliminate repetition (see line 179)

4. As a standard, please put a hyphen between “P value”, i.e., p-value.

This has been collected in the whole document

5. Line 168: Can the authors specify which interaction terms were assessed?

The variables assessed for interaction were included (see line 183-186)

6. Line 170-171: “the level of significance was set at 0.05 and the corresponding adjusted prevalence ratios (aPR), their corresponding 95% CIs and P values were computed” Similarly, there’s a repetition. I suggest removing the first “corresponding”.

The statement has been rephrased to eliminate repetition (see line 187)

7. Line 184: “We enrolled in 377…” remove “in”

“in” removed (see line 201)

8. Please ensure that all abbreviations in the tables are listed below that table.

All abbreviations mentioned in the table except those mentioned and defined in prior sections are highlighted in the table legends

9. I suggest restructuring lines 255–260 for improved logical flow. Specifically, the sentence “Whereas HIV testing has been associated with heightened anxiety and/or fear … help in reducing them at subsequent HIV testing” could be moved to precede the sentence starting “In the conventional HIV testing approaches, pre-test counselling delivered by health workers…”

The sentence has been moved as recommended (see line 278-280)

Reviewer #2: COMMENTS
The authors sought to determine the acceptability of HIVST among HIV high-risk AGYW living in urban communities in Uganda, using the Theoretical Framework of Acceptability (TFA).

Abstract
Nil

Introduction
1. The introduction cites HIV incidence data from 2021. It is recommended to reference the most recent UNAIDS estimates to strengthen the relevance of the study in the current context.

HIV incident data based on the UNAIDS 2024 report included with the appropriate citation (see line 54-55)

Methods
2. Please provide more detail on how participants were identified in the community using consecutive sampling. Specifically, clarify the locations and processes used to recruit potential participants. This information is critical for assessing whether the study population can be considered representative of the broader community.

The information on how VHTs conducted targeted mobilization of potential participants to selected areas including health facilities, youth corners, drop-in centers or community halls close by in the community is provided (see line 124-125)

3. Please clarify how the participant numbers were proportionately allocated using the 2023 population projections of AGYW in the study areas. Was this allocation based on a ratio between the areas, or was it part of the sample size calculation methodology? Providing this detail will help readers understand the sampling approach and its rationale.

After calculating the required sample size (N), the number to recruit from each study area (municipality/division) was apportioned according to the 2023 projected population of AGYW, so that those areas with higher number of AGYW according to the population projection (see line 120-122)

4. Clarify whether self-reported HIV status was considered in the eligibility criteria. To accurately assess the acceptability of HIV self-testing, it may be important to include only individuals who are not living with HIV. If this was not the case, explain the rationale for the chosen approach.

Self-reported HIV negative status or unknown HIV status was part of the inclusion criteria (see line 108-109)

5. The manuscript notes that information on HIVST kits and their use was provided to prospective participants. More detail on the specific content of this information would help clarify how it may have influenced participants' perceptions and the acceptability of the interventions.

The summary of specific information such as rationale for introduction, benefits and challenges as well as practical demonstration has been included (see line 132-134)

6. The questionnaire items are not included, making it difficult to assess how the questions align with the Theoretical Framework of Acceptability (TFA). Including the questionnaire as supporting information would enhance transparency and allow readers to evaluate the connection between the items and the framework.

The questionnaire has been included as a supplementary file.

7. The manuscript indicates that a Likert scale was used, but the meaning of responses 1–5 is not described. Clarifying what each response represents is essential to interpret what a score greater than 21 signifies. Including the study questionnaire as supporting information would further enhance transparency.

The study questionnaire has been provided as a supplementary file and cross referenced in-text as supplementary file 1 to guide readers on meaning of the Likert scale (see line 149)

8. The reported p-value cutoff of 0.2 for adjusted analysis appears high. Clarify how this threshold was determined and justify its use in the context of the study's analytical approach.

Usually a cutoff for a P value that is less stringent is used to accommodate for negative confounding effects some independent that may be masked in the unadjusted analysis.

Results

9. In the unadjusted analysis, “Had an STI in the past six months” and “Primary partner older by 5 years” had p-values <0.2 but were not included in the adjusted model, despite the description in the methods. Clarify the reason for excluding these variables from the adjusted analysis.

As described in the analysis plan, we used a backward elimination method, and some variables initially included for adjusted analysis dropped out of the final model presented because they were insignificant and yet worsened model performance. This procedure is enshrined in the backward elimination method of developing the prediction model.

10. Table 2 lists “condom use in the past 6 months” as a yes/no item, while the text refers to consistent condom use. This discrepancy makes it unclear whether the table reflects consistent use. Clarify the terminology or update the table to ensure alignment with the description in the text.

The correct terminology is “consistent condom use”. The table 2 descriptor had been edited to reflect this correction (see line 244)

11. In Table 2, the item “Used drug before or during sex” is ambiguous. Clarify whether this refers to recreational drugs, prescription medication, or another category to ensure accurate interpretation.

The drugs have been operationally defined as “recreational drugs” in the description of independent variables (see line 244)

12. The results section reports a median acceptability score, but the methods do not describe how this analysis was conducted. Include details of the approach in the methods to ensure consistency.

The descriptive analysis of the primary continuous outcome (acceptability scores) has been added in the data analysis section (see line 173-174)

13. Include the definition for “STI in the past 6 months” in the legend of Table 2, as described in the text. This will ensure the table can stand alone and be fully interpretable without referring to the main text.

The operational definition of STI and other variables included in the table are described under the section “independent variables”, and their inclusion on table will make it bulky (see line 163-164)

14. Partner characteristics are not presented in Table 1 or Table 2. Including the proportions for these characteristics would provide important context for interpreting the unadjusted and adjusted models and understanding their relevance to the study population.

The partner characteristics have been included in table 2 (last two variables)

Discussion
15. The discussion references a recent study among female university students in Uganda reporting similar findings but does not specify what those findings were. Including the key results from that study would substantiate the comparison and strengthen the argument.

The level of acceptability reported in the two studies have been reported explicitly (see line 263-264)

Grammar and formatting:
16. Check for consistency in spacing between words in text and in text reference.

Text font and spacing made consisted with PLOS guidelines

17. In the discussion section, try to minimize the overuse of transitional words such as however, whereas, furthermore, moreover, and nonetheless. Excessive use of these connectors can make the text feel repetitive and disrupt the natural flow. Aim to structure sentences so that the logical progression is clear without relying heavily on these linking words. This will help maintain readability and improve the overall coherence of your discussion.

Discussion revised to reduce on connectors.

Thank you for the very insightful comments
